# Enhancing Efficiency of Dye Sensitized Solar Cells by Coinage Metal Doping of Cyanidin-Silver Trimer Hybrids at TiO_2_ Support Based on Theoretical Study

**DOI:** 10.3390/nano14121034

**Published:** 2024-06-15

**Authors:** Margarita Bužančić Milosavljević, Martina Perić Bakulić, Željka Sanader Maršić, Antonija Mravak, Vlasta Bonačić-Koutecký

**Affiliations:** 1 Center of Excellence for Science and Technology-Integration of Mediterranean Region (STIM), Faculty of Science, University of Split, Ruđera Boškovića 33, 21000 Split, Croatia; margarita@stim.unist.hr; 2 Faculty of Chemistry and Technology, University of Split, Ruđera Boškovića 35, 21000 Split, Croatia; martina.peric-bakulic@ktf-split.hr; 3 Faculty of Science, University of Split, Ruđera Boškovića 33, 21000 Split, Croatia; zsm@pmfst.hr; 4 Interdisciplinary Center for Advanced Science and Technology (ICAST), University of Split, Meštrovićevo Šetalište 45, 21000 Split, Croatia; 5 Department of Chemistry, Humboldt Universität zu Berlin, Brook-Taylor-Strasse 2, 12489 Berlin, Germany

**Keywords:** bio-nano sensitizer, cyanidin-NC hybrid, DFT, TDDFT, light harvesting efficiency, coinage metal atoms

## Abstract

Identification of a natural-based sensitizer with optimal stability and efficiency for dye-sensitized solar cell (DSSC) application remains a challenging task. Previously, we proposed a new class of sensitizers based on bio-nano hybrids. These systems composed of natural cyanidin dyes interacting with silver nanoclusters (NCs) have demonstrated enhanced opto-electronic and photovoltaic properties. In this study, we explore the doping of silver nanocluster within a cyanidin-Ag_3_ hybrid employing Density Functional Theory (DFT) and its time-dependent counterpart (TDDFT). Specifically, we investigate the influence of coinage metal atoms (Au and Cu) on the properties of the cyanidin-Ag_3_ system. Our findings suggest that cyanidin-Ag_2_Au and cyanidin-AgAuCu emerge as the most promising candidates for improved light harvesting efficiency, increased two-photon absorption, and strong coupling to the TiO_2_ surface. These theoretical predictions suggest the viability of replacing larger silver NCs with heterometallic trimers such as Ag_2_Au or AgAuCu, presenting new avenues for utilizing bio-nano hybrids at the surface for DSSC application.

## 1. Introduction

The energy demand rises with increasing the global population and technological advancement [1,2]. The sun, an easily attainable, clean, renewable, and free energy resource, emerges as the optimal choice for generating sustainable energy. Consequently, solar harvesting technology holds the potential to supplant conventional energy sources [2,3]. According to the EU Market Outlook (2023), photovoltaic (PV) technology represents the fastest-growing energy production in European Union, with installed PV capacity increasing by more than 40% annually in recent years [4]. Additionally, there is an emphasis on advancing architecture-integrated photovoltaics to maximize solar harvesting efficiency. This involves integrating PV into smart buildings and surroundings to improve efficiency and achieve net-zero energy consumption [2,5]. Dye-sensitized solar cells (DSSCs) play an important role in the field of renewable energy by offering a promising solution, as a thin layered, flexible, and cost-effective technology. DSSCs were first proposed by O’Regan and Grätzel [6,7,8] as photochemical solar cells that imitate natural light absorption [9]. The fundamental idea behind DSSC lies in the interaction between dye and semiconductor, where dye absorbs light and sensitizes the transparent TiO_2_ semiconductor film. This allows electrons to be injected into the semiconductor’s conduction band, followed by regeneration of the dye through the electrolyte [10]. One of their key components is the dye sensitizer, which requires specific properties for efficient performance in DSSC. This includes high light harvesting efficiency (LHE) and suitable energy levels of the highest occupied (HOMO) and the lowest unoccupied (LUMO) molecular orbitals for effective charge transfer processes. Furthermore, it requires photostability and compatibility with other solar cell components, particularly anchoring to the semiconductor film [11]. In general, dye sensitizers can be classified [12] as organic [13,14], inorganic [15], metal-free organic dyes [16], and transition metal complexes [17]. Due to their non-toxicity, availability, and cost-effectiveness, natural dyes such as anthocyanidins [18] are an environmentally acceptable alternative to predominantly used Ru-based dyes [14,19,20,21]. However, the low efficiency of natural dyes limits their effectiveness [22], thus reducing overall DSSC performance. In response, various hybrid modifications of natural dyes have been researched so far [23,24,25,26,27].

In that context, we have previously computationally explored a new class of bio-nano sensitizers based on noble metal nanoclusters (NCs) interacting with natural (anthocyanidin) dyes [28]. In these bio-nano hybrid systems, nanoclusters introduce donor-acceptor properties shifting the HOMO and LUMO molecular orbitals in the desired direction, thus satisfying preconditions for successful electron injection and system regeneration [29]. The interaction between the excited states of nanoclusters and the excited π-π* states of aromatic rings in biomolecules enhances the absorption intensity within the bio-nano hybrid [30]. Consequently, combined DFT/TDDFT study has demonstrated improvements in photovoltaic properties, such as light harvesting efficiency (LHE) and driving force of electron injection (Δ$G^{inject}$), together with strong coupling to the TiO_2_ surface [28]. Among the examined bio-nano hybrids, those including Ag NCs with an even number of electrons (n = 2, 4, 8, 20) have demonstrated better stability, with cyanidin-(${\mathrm{Ag}}_{9}^{+}$) and cyanidin-(${\mathrm{Ag}}_{21}^{+}$) emerging as the most preferable model sensitizers.

Previously, DSSC efficiency has been enhanced through doping of nanostructured TiO_2_ by metal ions which induced electrical surface-state modifications [31]. In the present study, we theoretically explore the modification of the electronic and photophysical properties of bio-nano hybrid model sensitizers by hetero-metal atom doping. The justification for this strategy arises from the known potential of doping to tune the geometric and electronic properties of NCs while also improving their stability [32,33]. Specifically, we investigate the effects of substituting a single Ag atom in cyanidin-Ag_3_ with a coinage metal atom, such as gold or copper. In addition, to examine trimetallic hybrids, we also study the influence of mixed gold-copper doping. The selection of coinage metal atoms (Au, Cu) is motivated by their electronic configurations similar to that of Ag, leading to a closed shell for all doped systems. While silver atoms have a large s-d energy gap, gold and copper atoms possess significantly smaller s-d energy gaps [34,35]. Consequently, s electrons are crucial for bond formation in Ag clusters in contrast to d electrons in Au and Cu clusters [34]. Moreover, copper is a highly abundant, inexpensive metal and has familiar coordination chemistry [36]. Although gold is more expensive, it is nontoxic and shows promising linear and nonlinear optical properties when used as a dopant in ligated silver NCs [37].

Altogether, first, we elucidate how coinage metal doping influences the properties of hybrid systems. Second, we examine if systems with small doped NCs are viable alternatives to hybrids with larger silver clusters. For the computation of opto-electronic and photovoltaic properties, we adopt Density Functional Theory (DFT) and Time-Dependent Density Functional Theory (TDDFT) approaches. In addition to examining linear optical properties like one-photon absorption (OPA), the nonlinear optical properties, particularly two-photon absorption (TPA) are also investigated. Subsequently, we compare the obtained findings with those obtained from the reference monometallic hybrid system, cyanidin-Ag_3_. After the investigation of the doping effect in bio-Ag_3_ system, the role of TiO_2_ surface has been studied.

## 2. Computational Methods

The optimized geometry of the cyanidin-Ag_3_ was taken from our previous work as a reference model since this is the smallest investigated hybrid system with decent photovoltaic properties [28]. All cyanidin-Ag_2_Au and cyanidin-AgAuCu hybrids were optimized with the Perdew–Burke–Ernzerhof (PBE) [38] functional using the Gaussian 16 program [39]. The split valence polarization atomic basis set (SVP) [40] has been used for all atoms. Relativistic effective core potential (19-e^−^ RECP) [41] for silver and gold atoms has been employed. Previous studies on nanocluster-biomolecule hybrids [35] have demonstrated that this method accurately describes bio-nano hybrid systems containing silver, gold, and copper. To determine optical properties, the Coulomb-attenuated version of Becke’s three-parameter nonlocal exchange functional together with the Lee–Yang–Parr gradient-corrected correlation functional (CAM-B3LYP) [42] has been employed together with the same AO basis set. Nonlinear optical properties were obtained using the Dalton program [43,44]. TPA spectra and cross-sections (σ) [45] were calculated applying single residue or double residue quadratic response procedure [46,47].

The binding energy $E_{b}$ of the doped NC to the cyanidin dye is given by:

(1) $$E_{b}=E_{cyanidin-NC_{doped}}-\left(E_{cyanidin}+E_{NC_{doped}}\right)$$

with $E_{cyanidin-NC_{doped}}$ representing energy of the optimized doped hybrid, $E_{cyanidin}$ energy of cyanidin, and $E_{NC_{doped}}$ energy of the doped trimer.

The photovoltaic properties, such as light harvesting efficiency (LHE) is calculated according to Equation (2):

(2) $$LHE=1-10^{-f}$$

where *f* represents the oscillator strength of the maximum absorption. In addition, the driving force of electron injection (ΔG*^inject^*) is calculated according to Equation (3):

(3) $$\Delta G^{inject}=\left(-HOMO-\lambda_{max}\right)-ECB$$

where $\lambda_{max}$ is the maximum absorption energy and ECB experimental conduction band energy of TiO_2_ (−4 eV [7,48,49]).

The TiO_2_ surface model consisting of 30 TiO_2_ units capped with 12 hydrogens has been adopted from our earlier work [28]. The calculation of the structural and optical properties of the TiO_2_ model was performed at the same level of theory as for the hybrids, with the addition of W06 density fitting [40,50]. Different adsorption features strongly affect the binding of the hybrid to the surface and as a result, influence the efficacy of charge separation [12,51,52]. For that reason, we have previously modeled different positions of the hybrid relative to the surface and analyzed the electronic and optical properties of those structures [28]. The bio-nano hybrids are strongly anchored to the semiconductor model over cyanidin, showing significantly greater absorption intensities compared to other adsorption characteristics. Consequently, the modeling of the doped bio-nano hybrids’ anchoring to the semiconductor was carried out using the cyanidin dye. Adsorption energy *E_ads_* to TiO_2_ is given by:

(4) $$E_{ads}=E_{complex}-\left(E_{TiO_{2}}+E_{cyanidin-NC_{doped}}\right)$$

where $E_{complex}$ is the energy of the DFT optimized adsorbed hybrid and $E_{TiO_{2}}$ energy of the TiO_2_ model.

## 3. Results and Discussion

### 3.1. Structural and Electronic Properties of Cyanidin-Trimer Hybrids

**Figure 1:**
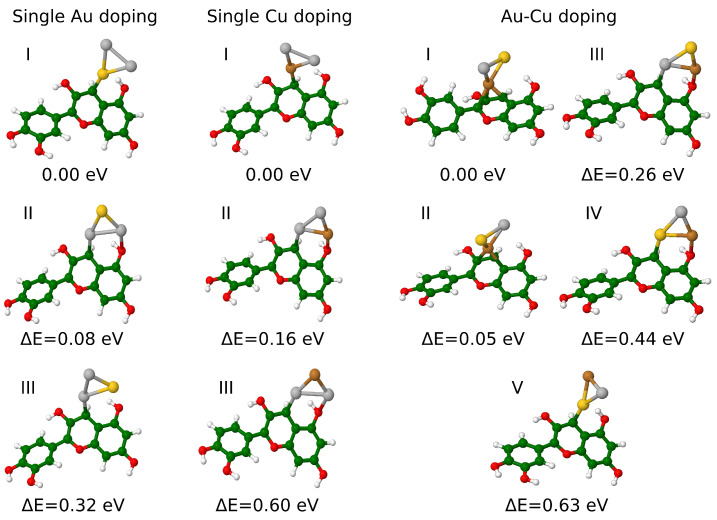
Isomers of cyanidin-Ag_2_Au, cyanidin-Ag_2_Cu and cyanidin-AgAuCu optimized at PBE/def2-SVP level of theory. Numbers I–V indicate the order of isomers. Ag, Au, Cu, C, O, and H atoms are depicted in grey, yellow, brown, green, red, and white.

The single Au-doped structures were obtained by substituting one Ag atom at various positions in Ag_3_^+^ with a single Au atom, resulting in three different cyanidin-Ag_2_Au isomers. Similarly, the single Cu-doped structures were obtained by exchanging one Ag by one Cu. Additionally, to investigate the effects of mixed Au-Cu doping, cyanidin-AgAuCu systems were examined. Here, the two Ag atoms were simultaneously replaced by one Au and one Cu atom, yielding six possible combinations. During optimization, the two structures reached the same minimum and were consequently considered as one, resulting in five Au-Cu-doped isomers. Figure 1 illustrates the obtained isomers and their corresponding relative energies. Note that Ag_3_^+^ has a closed shell, so the replacement of one or two Ag atoms by the coinage metal atom (which is in the same periodic group) results in a closed shell system.

An illustration of bonding between the cluster and the cyanidin, as well as the bond length analysis within each doped trimer isomer, is presented in Figure 1 and Table S1, respectively. The lowest energy isomer of cyanidin-Ag_2_Au forms a single Au-C bond between the trimer and the dye. By comparison, the lowest energy isomer of Cu-doped systems forms a single Cu-C bond. In contrast, in cyanidin-AgAuCu isomer I, the cluster is bound to the dye through three Cu-C bonds.

This diverse bonding between the trimer and the dye significantly changes the stability of the systems as evident by the energy difference between isomers. Since the doping of Ag_3_ generally affects their stability [32], the binding energies ($E_{b}$) of the doped trimer bound to the cyanidin dye have also been calculated (Table 1) and compared to $E_{b}$ of cyanidin-Ag_3_ (−12.45 eV) [28]. Overall, the binding energies of the doped systems are increased by ∼0.2 up to ∼1.1 eV, suggesting that the doping with single Au or Cu and mixed Au-Cu significantly enhances the stability of the hybrids. Considering that Ag NCs often suffer from lower stability [32], doping could be an important strategy for the future applicability of bio-nano hybrid systems.

Furthermore, the charge transfer between different atoms within doped trimers plays a key role in their structural and electronic properties [34]. For this purpose, Mulliken charge analysis has been performed, revealing variation in NC/dye charge transfers between different isomers of the same doping group. The sum of the charges on the single Au-doped trimers ranges from −0.19 to 0.07, for Cu-doped trimers from 0.11 to 0.2, and mixed Au-Cu-doped from −0.24 to 0.08 (cf. Table 1). Notably, Au atoms exhibit negative charges in all Au-doped and mixed Au-Cu-doped systems, consistent with previous findings that Au withdraws electrons from Ag in Au-Ag NCs [34].

### 3.2. Linear Optical and Photovoltaic Properties of Cyanidin-Trimer Hybrids

Appropriate positioning of the HOMO and LUMO levels is crucial to meet the prerequisites for successful electron injection and system regeneration [10]. Our previous study on bio-nano hybrids has demonstrated that the addition of Ag NCs to anthocyanidin dyes effectively shifts the HOMO and LUMO levels in the desired direction [28]. Accordingly, analysis of HOMO and LUMO levels shows that all of the doped isomers have favorably positioned HOMO and LUMO levels. HOMO is lying below I^−^/I_3_^−^ redox potential (−4.6 eV [53] vs. vacuum) and LUMO above TiO_2_ conduction band, thus satisfying the requirement for an efficient sensitizer. The HOMO, LUMO values, maximum absorptions, and the key photovoltaic properties are listed in Table 2.

Due to a connection between the strong and broad sensitizer absorption in visible and near-infrared (NIR) and the photocurrent generation [10], we have examined the UV-VIS spectra for all modeled systems. Figures S1–S3 provide orbital analysis and comparison of the absorption spectrum of cyanidin-Ag_3_ with those of isomers within the Au, Cu, and Au-Cu doping groups, respectively.

**Figure 2:**
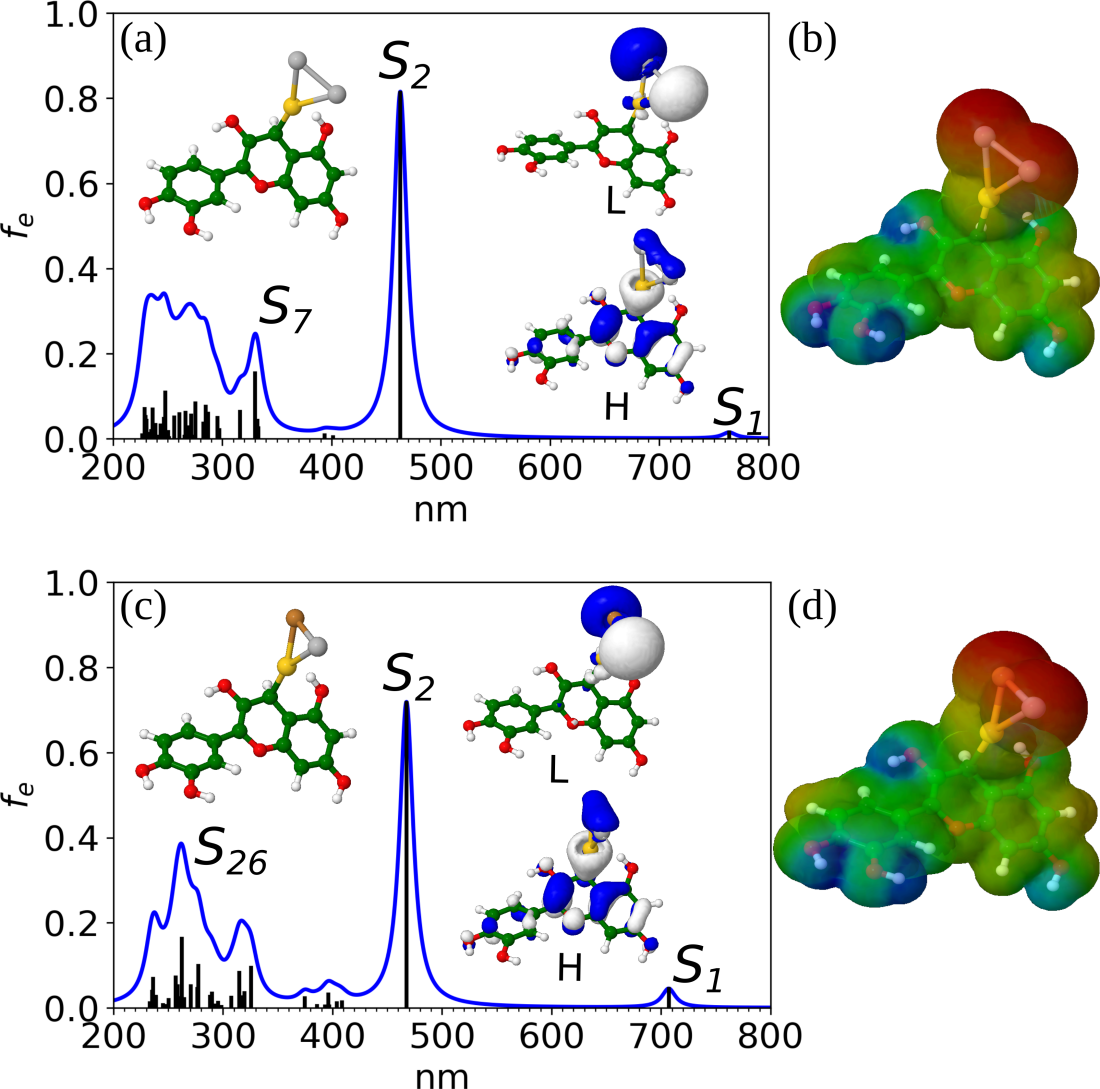
One-photon absorption spectra of (**a**) cyanidin-Ag_2_Au (isomer I) and (**c**) cyanidin-AgAuCu (isomer V) at CAM-B3LYP/def2-SVP level of theory. The inset figures represent HOMO and LUMO orbitals. (**b**,**d**) Molecular electrostatic potential (MEP) surfaces of corresponding doped systems with red color representing electron-rich and blue color electron-poor area. For atom color legend please refer to the caption of Figure 1.

Generally, absorption spectra of the doped hybrids exhibit characteristic features similar to the hybrid with monometallic Ag NC. Specifically, two distinct transition regions can be observed, one around 300 and the second around 500 nm. For all systems, the first peak characterized by very low intensity corresponds to the HOMO → LUMO transition. Orbital analysis of the doped model systems reveals that the HOMO is predominantly delocalized over cyanidin and one trimer atom directly connected to the carbon atom of the dye (cf. Figures S1 and S2). By comparison, the doped systems containing Au bound to the dye through a single bond exhibit delocalization over the entire structure, including both the trimer and the dye (cf. Figure 2a,c). In contrast, the LUMO is delocalized only on the trimer. The strongest transition is S_2_ for most of the isomers, representing the HOMO → LUMO + 1 transition. The latter orbital is delocalized over the trimer and the area around its connection with the dye.

As shown in Figures S1 and S3, and Table 2, the overall largest maximum absorption is present in the spectra of cyanidin-Ag_2_Au I and cyanidin-AgAuCu V. Their maximum absorption is almost at the same wavelength as in the case of the cyanidin-Ag_3_ (∼460 nm, cf. Table 2, Figure 2a,c), but the oscillator strength is increased from 0.57 to 0.81 and 0.72, respectively. This has direct consequences on the calculation of the LHE which is the highest for the Au-doped isomer I (0.85) while the second best LHE corresponds to isomer V of Au-Cu-doped systems (0.81). These values are approximately the same as those of cyanidin-(${\mathrm{Ag}}_{9}^{+}$) and cyanidin-(${\mathrm{Ag}}_{21}^{+}$) which were identified as optimal sensitizers in our previous study [28]. This shows the potential of replacing larger Ag NCs in bio-nano hybrid systems with smaller doped trimers, opening new possibilities for designing cost-effective bio-nano-based sensitizers with improved stability and light harvesting efficiency.

The two systems with the largest LHE have trimer bound to cyanidin via a single Au-C bond. Overlapping these two structures shows that dihedral angles between the Au-C bond and the indoline plane (118° for the Au-doped and 113° for the Au-Cu-doped hybrid) are nearly the same. Interestingly, in both cases the sum of Mulliken charges on the doped trimer is 0.02, demonstrating the importance of the charge and its redistribution within the different metal atoms on the LHE. The type of the dopant atom(s), as well as its position, affects the LHE improvement.

Following earlier studies, we have also examined the electronic distribution of the cyanidin-Ag_2_Au I and cyanidin-AgAuCu V by visualizing their molecular electrostatic potential (MEP) surfaces, presented in Figure 2b,d. In agreement with the previous findings, two distinct regions are present; the electron-rich area on the trimer (the donor) and the electron-poor area on the cyanidin dye (the acceptor).

Further analysis of isomers II and III in the Au-doped group reveals LHE values similar to the reference ones. The Cu-doping isomers generally exhibit comparable but slightly lower LHE, with isomer II demonstrating the highest value within the group (0.7). The mixed Au-Cu doping group exhibits diverse LHE values, ranging from 0.41 to 0.81.

In addition to the LHE, Δ$G^{inject}$ also indicates if the selected models are suitable for DSSC application. The negative Δ$G^{inject}$, observed in all doped systems, suggesting that the electron injection process is spontaneous for all examined systems.

### 3.3. Nonlinear Optical Properties of Cyanidin-Trimers Hybrids

While conventional photovoltaic materials primarily rely on OPA to generate electron-hole pairs per absorbed photon, TPA enables the creation of such pairs through the simultaneous absorption of two photons. This mechanism substantially enhances the absorption cross-section of materials in DSSCs, allowing for the capture of a greater number of light photons across a wider spectrum of wavelengths. Consequently, it has the potential to increase light harvesting efficiency under low intensity light and improve the overall efficiency of the photovoltaic device [54,55,56]. TPA spectra for the monometallic cyanidin-Ag_3_ model, as well as the dopant models cyanidin-Ag_2_Au (isomer I) and cyanidin-AgAuCu (isomer V), are illustrated (cf. Figure S4). For the cyanidin-Ag_3_ model, a substantial cross-section is already observed in the visible part of the spectra, around 460 nm (cf. Figure S4a). Doping with gold significantly enhances the intensity of the cross-section, highlighting the donor-acceptor nature and charge transfer between cyanidin and the heterometallic cluster in the gold-doped bio-nano hybrid (cf. Figure S4b). Additionally, when copper is added to the gold-doped bio-nano hybrid, a prominent peak emerges, surpassing the others in intensity (cf. Figure S4c). Analysis of the molecular orbitals for the last model demonstrates that the HOMO and LUMO for S_39_ excited state are concentrated solely on the Ag-Au-Cu nanocluster. Doping with Au and Cu induces enhanced values of cross-sections around 460 nm, due to resonance between one-photon and two-photon excited states (cf. Figure S5). The optical properties of Au, Ag, and Cu atoms differ because of variations in their electronic configurations, particularly the occupancy of their s and d orbitals [57,58,59,60]. These electronic configurations determine how the atoms interact with light. Previous studies have established that gold with the smaller s-d energy gap [34], has a more pronounced relativistic effect compared to silver, allowing the d electrons to participate in bonding.

### 3.4. Cyanidin-Trimer Sensitizer Anchored on a TiO_2_ Model Surface

**Figure 3:**
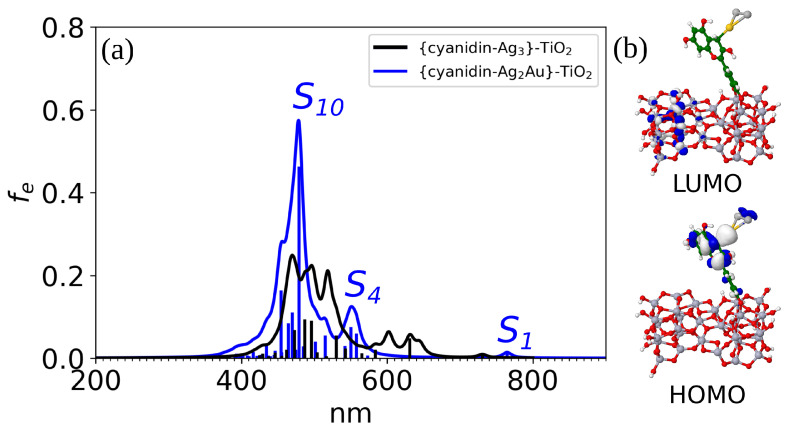
(**a**) Absorption spectrum of {cyanidin-Ag_2_Au}-TiO_2_ at CAM-B3LYP/def2-SVP level of theory. (**b**) Visualization of HOMO and LUMO orbitals; HOMO orbital is delocalized entirely at the bio-nano hybrid and LUMO at the TiO_2_ model. Ti atoms are depicted in lilac color.

We have selected cyanidin-Ag_2_Au (isomer I) with the largest LHE and anchored it to a semiconductor model in order to create a realistic system of the doped dye adsorbed on a TiO_2_ surface. The bonding between the TiO_2_ model over two Ti-O bonds ensures a strong binding energy of −1.9 eV. Even though the region of maximum absorption is at the approximately same wavelength (∼480 nm), the absorption spectrum of the supported doped hybrid reveals much stronger intensities compared to anchored cyanidin-Ag_3_ (Figure 3a). The HOMO is localized over the whole cyanidin-Ag_2_Au I and it is energetically at the same position as the free doped hybrid, while the LUMO is completely delocalized on the TiO_2_ surface model (Figure 3b). Also, the energy level of the LUMO is close to the LUMO of the dye. The spatial distribution of the HOMO, LUMO orbitals illustrates the charge transfer between the dye and the semiconductor model. The main contributions to the strongest peak include transitions from the bio-nano hybrid to the trimer and semiconductor model (LUMO + 43) and entirely to the semiconductor (LUMO + 6, LUMO + 18) (cf. Table S2 and Figure S6).

## 4. Conclusions

In this contribution, we have theoretically investigated the doping strategy for tuning the opto-electronic and photovoltaic properties of bio-nano hybrid systems at TiO_2_ surface. Extensive analysis of single Au, single Cu, and mixed Au-Cu doping of Ag NC within the hybrid revealed that the isomers cyanidin-Ag_2_Au I and cyanidin-AgAuCu V exhibit the best LHE value while also satisfying other preconditions for efficient DSSC sensitizer. Overall, these systems share similar structural properties and the same net trimer charge. This is the result of charge redistribution between the dopant atom(s) and the Ag which plays a key role in the structural properties of heterometallic trimer. Furthermore, the origin of their superior opto-electronic and photovoltaic properties is most likely due to the influence of the different s-d energy gaps for Ag and Au/Cu. Remarkably, TPA calculations on isomers I and V exhibit substantially higher intensities compared to the monometallic cyanidin-trimer hybrid, with cyanidin-AgAuCu having the largest cross-section. In addition, a successful electron injection is ensured through strong binding to the TiO_2_ semiconductor with the anchored system revealing significantly enhanced calculated absorption compared to our previous studies. Thus, such systems could substitute previously predicted bio-nano hybrids based on larger Ag_9_^+^ and Ag_21_^+^ clusters. Our preliminary studies on single Au-doped cyanidin-Ag_8_Au have shown no improvements in photovoltaic properties. Hence, an effect of single Au and mixed Au/Cu doping is pronounced only on smaller cyanidin-trimer hybrids. The open question is whether doping with two or more atoms would produce the same effect on larger NCs.

Since low stability in silver nanoclusters (Ag NCs) is present, strategies such as coinage metal doping emerge as an important way for enhancing the stability of bio-nano hybrid systems at surfaces. The cost-effective way to design new bio-nano-based sensitizers with improved stability and light harvesting efficiency is the replacement of larger Ag NCs in bio-nano hybrid systems by smaller doped trimers. Future experimental preparation of such bio-nano sensitizers should connect the theory and application, with the ultimate goal of designing energy harvesting systems with superior properties.

## Figures and Tables

**Table 1:** Binding energies of the doped NCs to the dye and the analysis of Mulliken charges on the doped-NCs within bio-nano hybrid systems. The order of silver atoms (Ag_1_, Ag_2_) is chosen starting from Au atoms clockwise.

	**Au Doping**				
	**$E_{b}$ [eV]**	**Net Trimer Charge**	**Au Charge**	**Ag_1_ Charge**	**Ag_2_ Charge**
Isomer I	−12.95	0.02	−0.20	0.09	0.13
Isomer II	−12.87	0.07	−0.07	0.15	−0.01
Isomer III	−12.63	−0.19	−0.28	0.05	0.04
	**Cu Doping**				
	**$E_{b}$ [eV]**	**Net Trimer Charge**	**Cu Charge**	**Ag_1_ Charge**	**Ag_2_ Charge**
Isomer I	−12.77	0.20	0.17	0.01	0.02
Isomer II	−12.61	0.11	0.20	−0.03	−0.06
Isomer III	−12.17	0.12	0.09	0.09	−0.06
	**Au-Cu Doping**				
	**$E_{b}$ [eV]**	**Net Trimer Charge**	**Au Charge**	**Cu Charge**	**Ag Charge**
Isomer I	−13.56	−0.24	−0.22	−0.07	0.05
Isomer II	−13.50	−0.06	−0.21	0.15	−0.001
Isomer III	−13.29	0.08	−0.13	0.22	−0.01
Isomer IV	−13.12	0.02	−0.10	0.20	−0.08
Isomer V	−12.92	0.02	−0.17	0.21	−0.02

**Table 2:** Calculated HOMO, LUMO levels, wavelengths ($\lambda_{max}$) and oscillator strengths ($f_{e}$) of maximum absorption, LHE, Δ$G^{inject}$ and HOMO-LUMO gaps at CAM-B3LYP/def2-SVP level of theory.

	HOMO [eV]	LUMO [eV]	$\lambda_{\max}$ [nm]	$f_{e}$	LHE	ΔG*^inject^* [eV]	HOMO-LUMO
cyanidin-Ag_3_ *	−5.35	−1.65	467	0.57	0.73	−1.30	3.70
cyanidin-Ag_9_ *	−5.28	−1.68	311	0.71	0.80	−2.71	3.60
cyanidin-Ag_21_ *	−4.82	−2.52	332	0.68	0.79	−2.91	2.30
cyanidin-Ag_2_Au I	−5.58	−2.08	463	0.81	0.85	−1.10	3.50
cyanidin-Ag_2_Au II	−5.52	−1.71	435	0.52	0.70	−1.33	3.81
cyanidin-Ag_2_Au III	−5.29	−2.53	518	0.46	0.65	−1.10	2.77
cyanidin-Ag_2_Cu I	−5.51	−1.84	440	0.37	0.57	−1.31	3.67
cyanidin-Ag_2_Cu II	−5.30	−0.98	444	0.52	0.70	−1.49	4.33
cyanidin-Ag_2_Cu III	−5.29	−1.72	457	0.51	0.69	−1.42	3.57
cyanidin-AgAuCu I	−5.68	−1.96	449	0.23	0.41	−0.26	3.72
cyanidin-AgAuCu II	−5.42	−2.12	228	0.27	0.46	−4.01	3.29
cyanidin-AgAuCu III	−5.51	−0.86	425	0.45	0.65	−1.41	4.65
cyanidin-AgAuCu IV	−5.53	−1.06	419	0.62	0.76	−1.43	4.47
cyanidin-AgAuCu V	−5.54	−1.89	468	0.72	0.81	−1.11	3.65

* Ref [28].

## Data Availability

The original contributions presented in the study are included in the article and Supplementary Materials, further inquiries can be directed to the corresponding author.

## Supplementary Materials

The following supporting information can be downloaded at: https://www.mdpi.com/article/10.3390/nano14121034/s1, Figure S1: Comparison of absorption spectra of cyanidin-Ag_2_Au isomers and cyanidin-Ag_3_ at CAM-B3LYP/def2-SVP level of theory; Figure S2: Comparison of OPA spectra of cyanidin-Ag_2_Cu isomers and cyanidin-Ag_3_ at CAM-B3LYP/def2-SVP level of theory; Figure S3: Comparison of OPA spectra of cyanidin-AgAuCu isomers and cyanidin-Ag_3_ at CAM-B3LYP/def2-SVP level of theory; Figure S4: TPA spectra obtained by TDDFT at CAM-B3LYP/def2-SVP level of theory for cyanidin-Ag_3_, cyanidin-Ag_2_Au isomer I, and cyanidin-AgAuCu isomer V; Figure S5: Energies of one-photon OPA and two-photon TPA illustrating the resonance in cyanidin-Ag_3_, cyanidin-Ag_2_Au isomer I, and cyanidin-AgAuCu isomer V; Figure S6: Absorption spectrum of {cyanidin-Ag_2_Au}-TiO_2_ at CAM-B3LYP/def2-SVP level of theory with the main transitions; Table S1: Analysis of bond lengths of the doped NCs within bio-nano hybrid systems; Table S2: Analysis of the key excited states of {cyanidin-Ag_2_Au}-TiO_2_ at CAM-B3LYP/def2-SVP level of theory.
