# Supplementary material for: Enhancing Efficiency of Dye Sensitized Solar Cells by Coinage Metal Doping of Cyanidin-Silver Trimer Hybrids at TiO2 Support Based on Theoretical Study
Source: Nanomaterials (Basel). 2024 Jun 15;14(12):1034. doi: 10.3390/nano14121034 (PMC11206320; doi:10.3390/nano14121034)
Supplement: Supplementary file 1 [file nanomaterials-14-01034-s001.zip › nanomaterials-3050295-supplementary.pdf]

Supplementary Materials: Enhancing Efficiency of Dye Sensitized Solar Cells by Coinage Metal Doping of Cyanidin-Silver Trimer Hybrids at TiO_2 Support Based on Theoretical Study

Margarita Bužančić Milosavljević [1], Martina Perić Bakulić [2], Željka Sanader Maršić [3], Antonija Mravak [3,*] and Vlasta Bonačić-Koutecký [1,4,5,*]

Table S1. Analysis of bond lengths of the doped NCs within bio-nano hybrid systems. The order of silver atoms (Ag_1, Ag_2) is chosen starting from Au atoms clockwise.

		**Au doping**	
	**Au-Ag$_1$ [Å]**	**Au-Ag$_2$ [Å]**	**Ag$_1$-Ag$_2$ [Å]**
Isomer I	2.79	2.78	2.61
Isomer II	2.64	2.77	2.72
Isomer III	2.73	2.60	2.87
		**Cu doping**	
	**Cu-Ag$_1$ [Å]**	**Cu-Ag$_2$ [Å]**	**Ag$_1$-Ag$_2$ [Å]**
Isomer I	2.64	2.52	2.64
Isomer II	2.52	2.45	2.81
Isomer III	2.43	2.57	2.76
		**Au-Cu doping**	
	**Au-Ag [Å]**	**Au-Cu [Å]**	**Ag-Cu [Å]**
Isomer I	2.82	2.47	2.27
Isomer II	2.83	2.48	2.47
Isomer III	2.77	2.44	2.53
Isomer IV	2.79	2.52	2.46
Isomer V	2.90	2.48	2.42

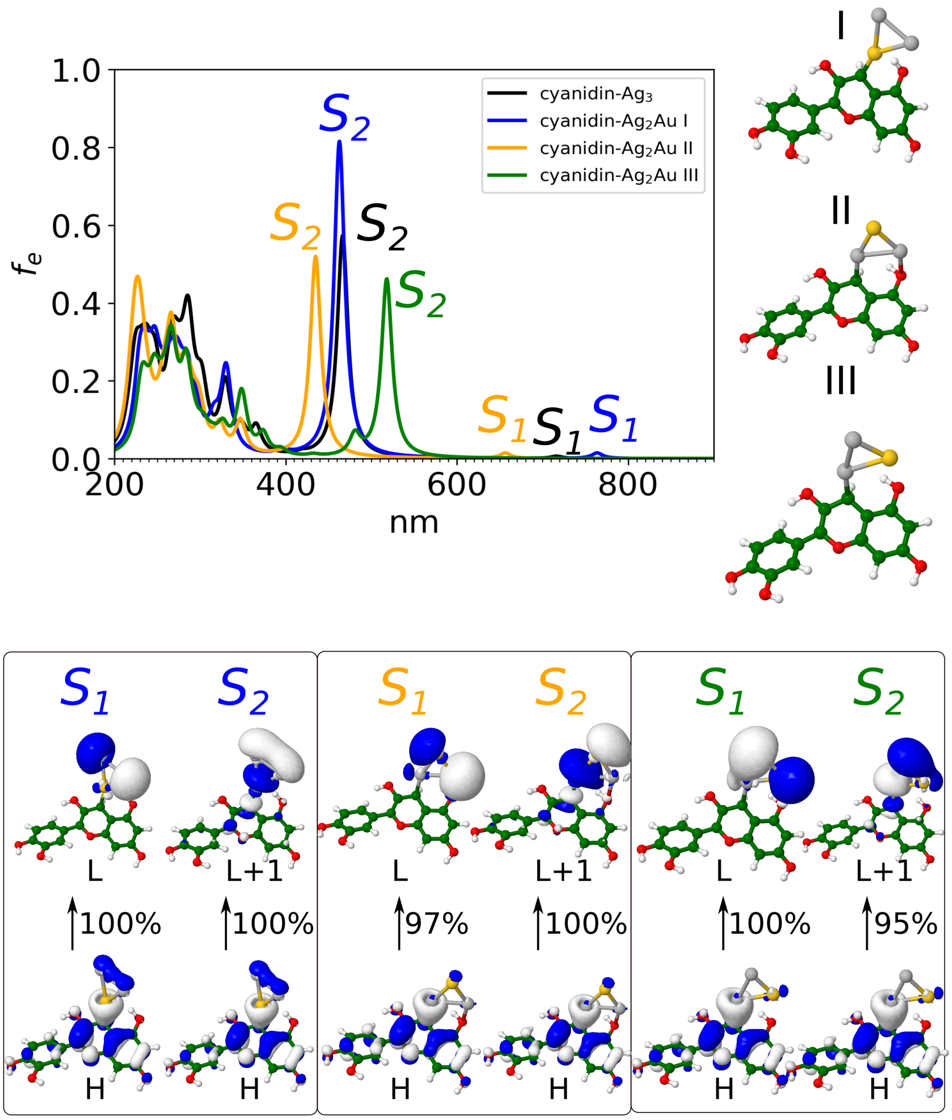

Figure S1. Comparison of absorption spectra of cyanidin-Ag_2Au isomers and cyanidin-Ag_3 at CAM-B3LYP/def2-SVP level of theory. The structures of Au-doped isomers optimized at the PBE/def2-SVP level of theory are shown on the right. The transitions with orbitals analysis corresponding to S_1 and S_2 states are shown below.

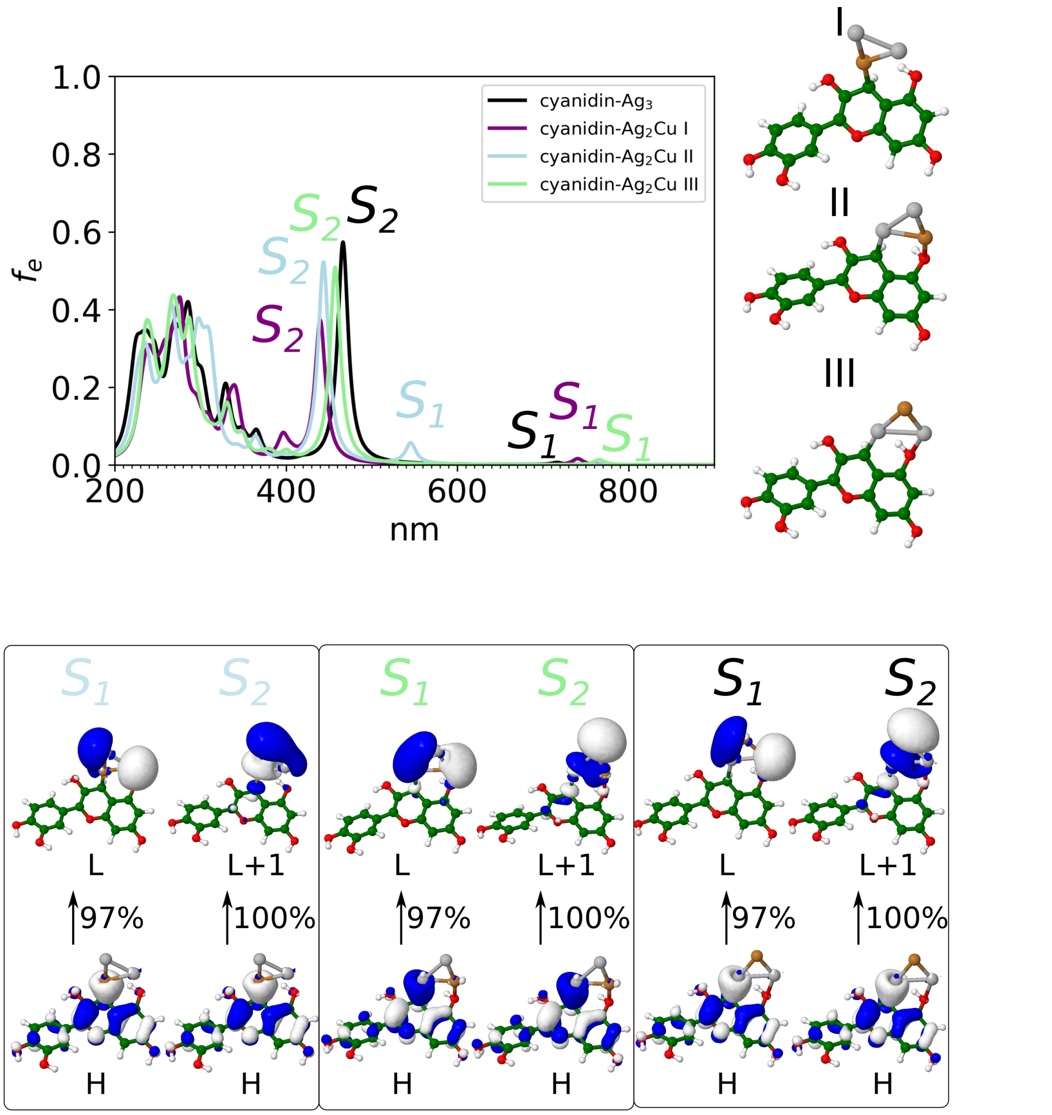

Figure S2. Comparison of OPA spectra of cyanidin-Ag_2Cu isomers and cyanidin-Ag_3 at CAM-B3LYP/def2-SVP level of theory. The structures of Cu-doped isomers optimized at the PBE/def2-SVP level of theory are shown on the right. The transitions with orbitals analysis corresponding to S_1 and S_2 states are shown below.

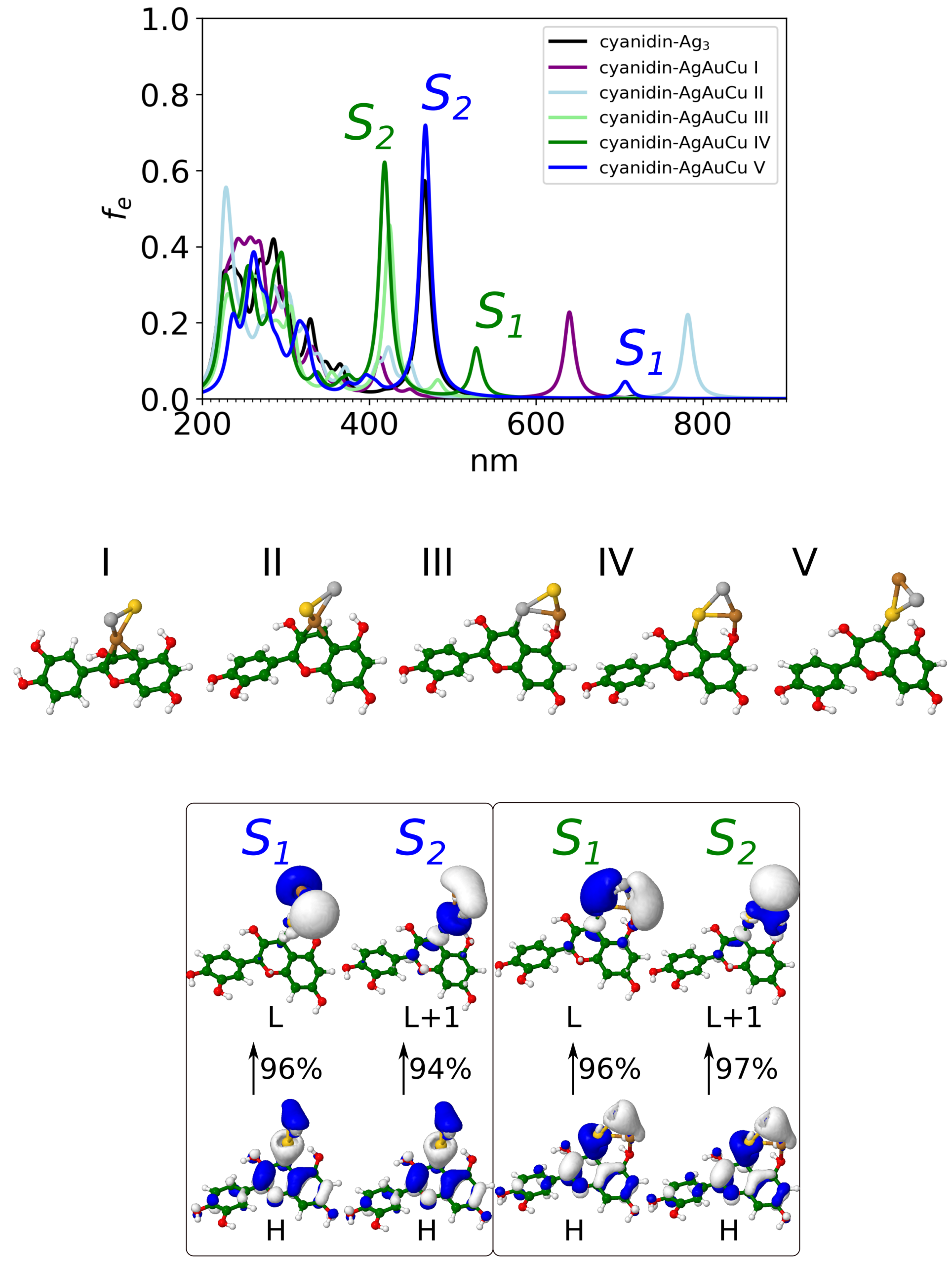

Figure S3. Comparison of OPA spectra of cyanidin-AgAuCu isomers and cyanidin-Ag_3 at CAM-B3LYP/def2-SVP level of theory. The structures of Au-Cu-doped isomers optimized at the PBE/def2-SVP and transitions with orbitals analysis corresponding to S_1 and S_2 states are shown below.

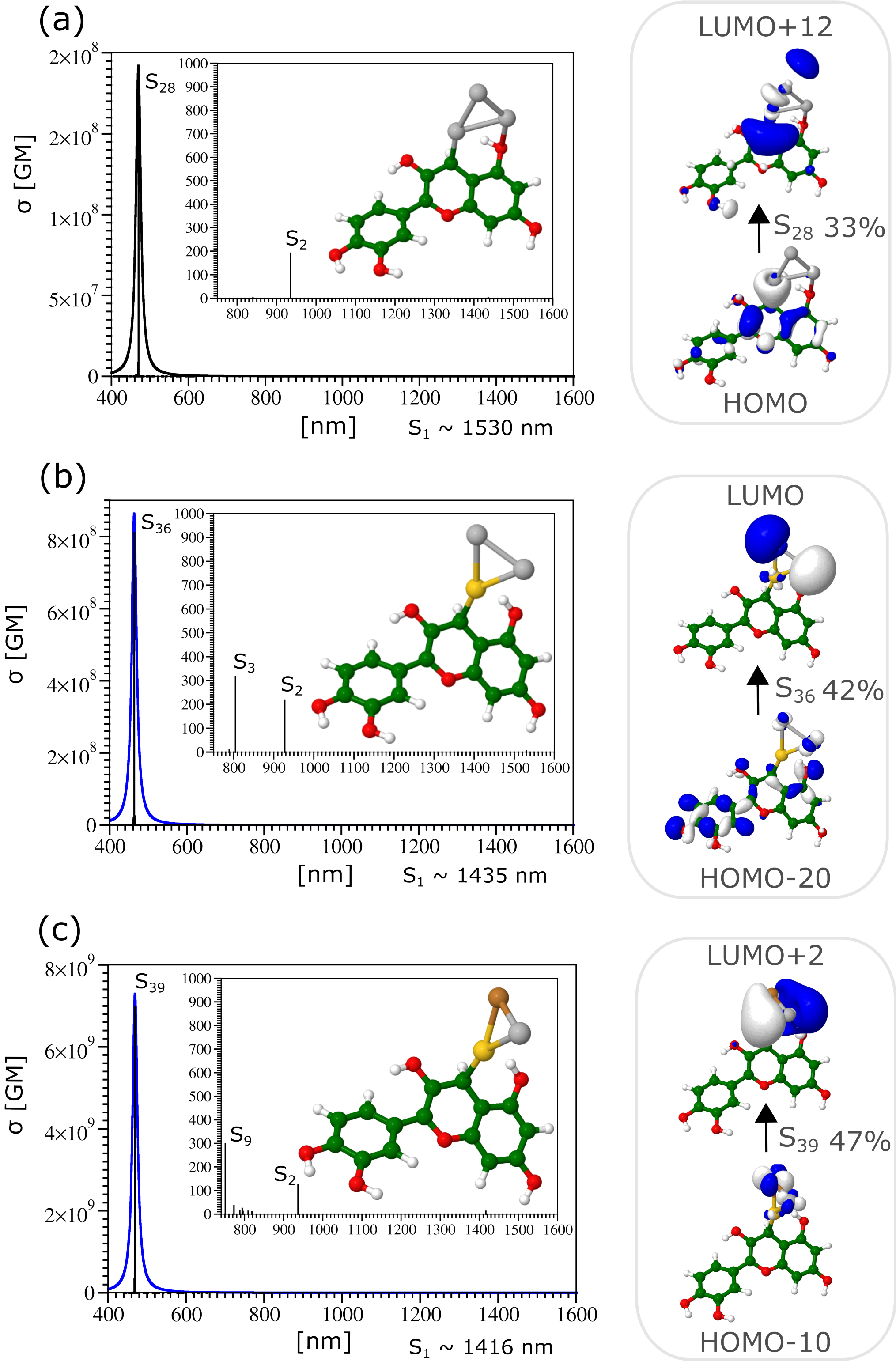

Figure S4. TPA spectra obtained by TDDFT at CAM-B3LYP/def2-SVP level of theory for (a) cyanidin-Ag_3, (b) cyanidin-Ag_2Au isomer I, and (c) cyanidin-AgAuCu isomer V. TPA cross sections for states in IR/NIR regime are presented as insets. The molecular orbital analysis for leading excitations is shown on the right.

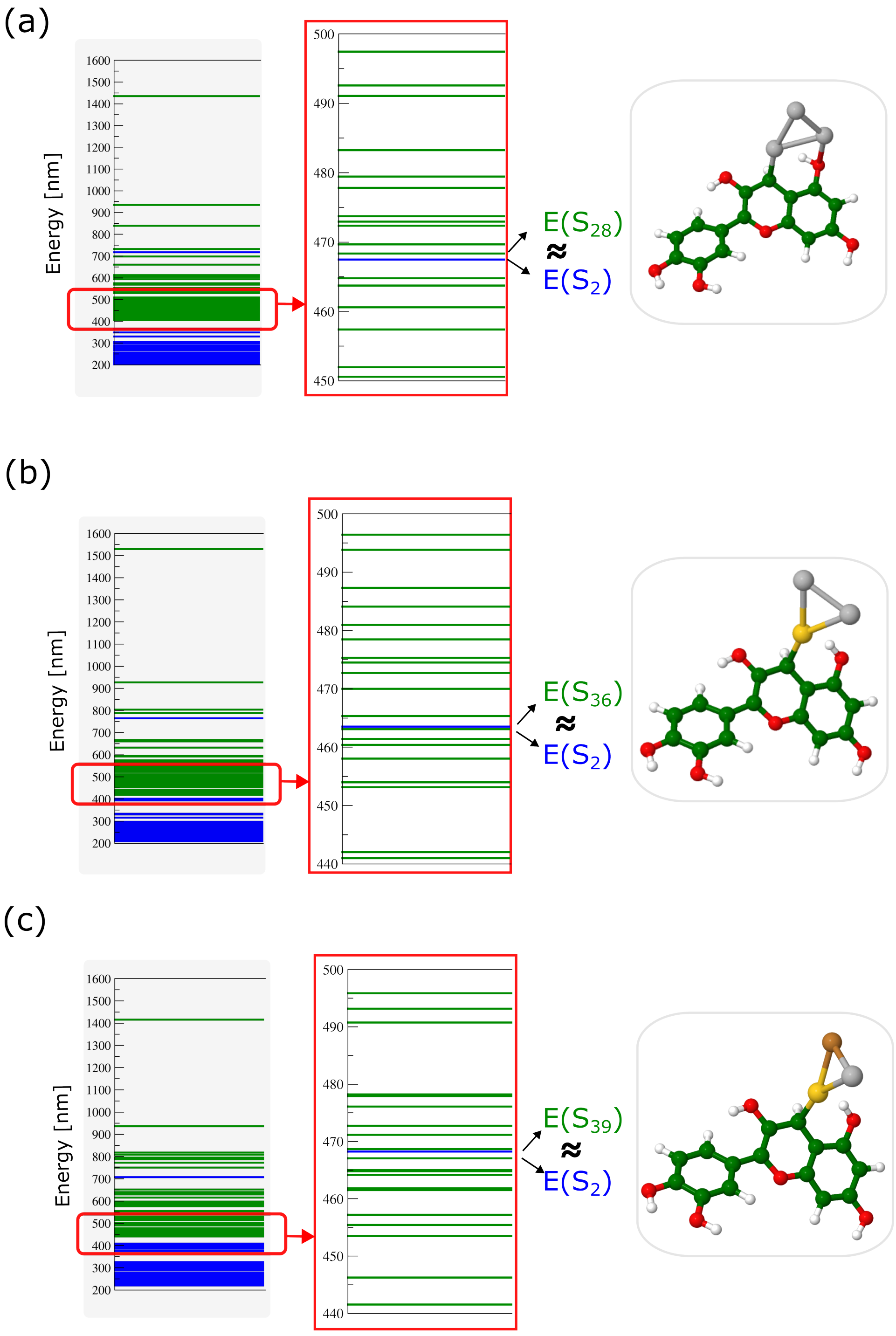

Figure S5. Energies of one-photon OPA (blue) and two-photon TPA (green) states illustrating the resonance between (a) cyanidin-Ag_3, (b) cyanidin-Ag_2Au isomer I, and (c) cyanidin-AgAuCu isomer V.

Table S2. Analysis of the key excited states of {cyanidin-Ag_2Au}-TiO_2 at CAM-B3LYP/def2-SVP level of theory.

Excited state	λ[nm]	f_e	Transitions
S_1	765	0.0134	H → L+9 (100%)
S_4	550	0.0718	H → L+1 (27%)
			H → L+13 (21%)
S_{10}	479	0.4592	H → L+43 (25%)
			H → L+6 (25%)
			H → L+18 (11%)

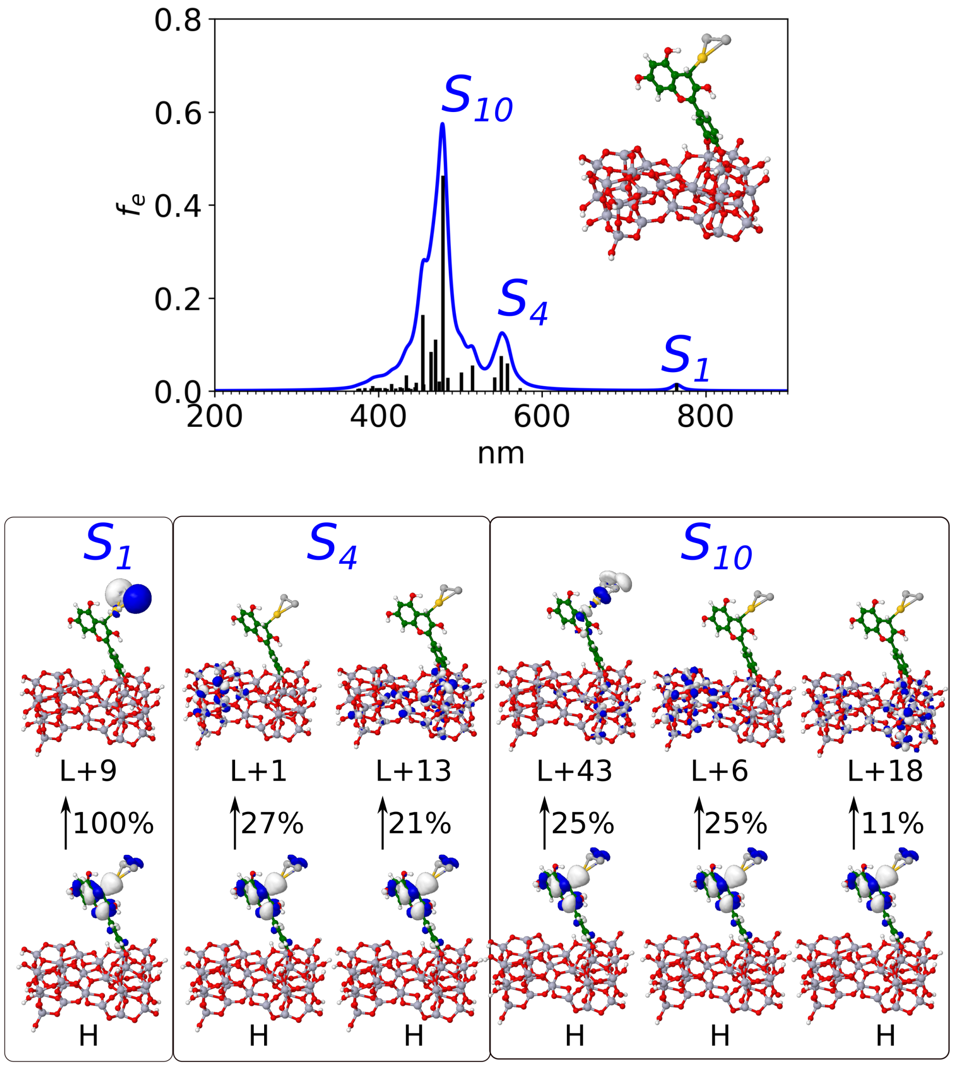

Figure S6. Absorption spectrum of {cyanidin-Ag_2Au}-TiO_2 at CAM-B3LYP/def2-SVP level of theory with the main transitions.
